# Supplementary material for: Increased Expression of TLR4 in Circulating CD4+T Cells in Patients with Allergic Conjunctivitis and In Vitro Attenuation of Th2 Inflammatory Response by Alpha-MSH
Source: Int J Mol Sci. 2020 Oct 23;21(21):7861. doi: 10.3390/ijms21217861 (PMC7672642; doi:10.3390/ijms21217861)
Supplement: Supplementary file 1 [file ijms-21-07861-s001.zip › Figure S1.pdf]

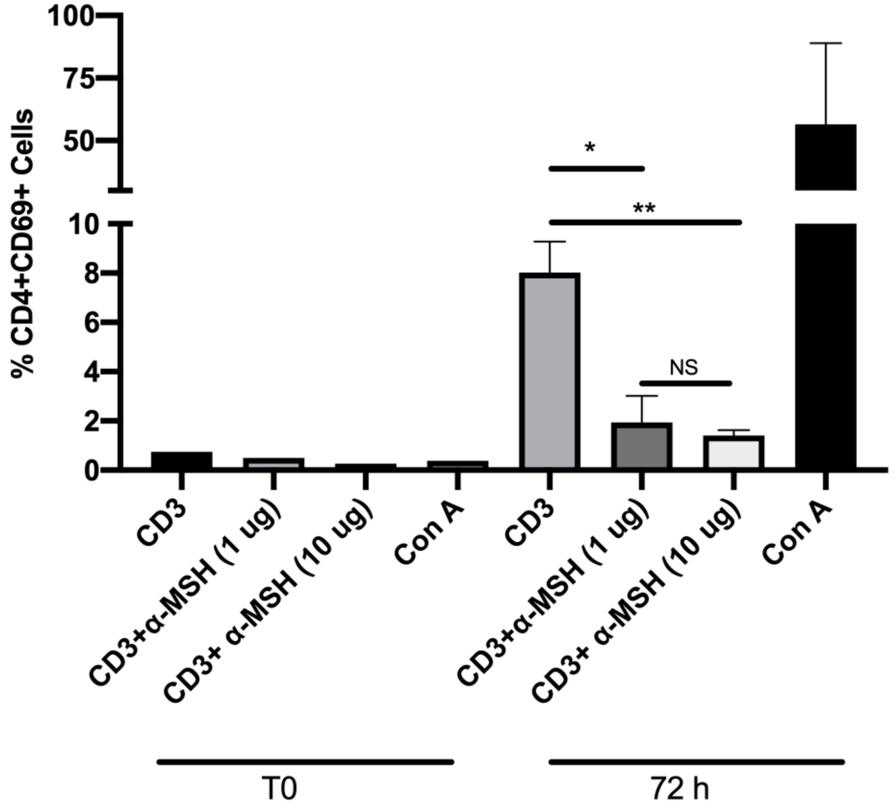

Figure S1. CD69 expression on CD4 T cells co-cultured with CD3 and α-MSH. To determine the optimum dose of α-MSH, we obtained blood samples from healthy donors ($n = 3$). PBMC were isolated, and cultured in 96-well flat-bottomed cell culture plates (Costar, Cambridge, MA, USA) pretreated with anti-CD3 (BioLegend, San Diego, CA, USA) at 2 × 10^5 cells/well in RPMI-1640 medium supplemented with 1 mM sodium pyruvate, 2 mM L-glutamine, and incubated at 37 °C in a 5% CO_2 humidified chamber After 24 h, the culture medium was removed, and a fresh culture medium supplemented with 10% heat-inactivated fetal calf serum and α-MSH at 1 mg/mL or 10 mg/mL was added (Time 0, T0). After 72 h, the cells were harvested and processed to evaluate T cell activation with CD69 expression on CD4+ T cells by flow cytometry.The harvested cells were analyzed for CD69 expression (coupled to FITC) on CD4 T cells (coupled to phycoerythrin Cy-5 tandem conjugate, PeCy5) on a BD FACSVerse™ (BD Biosciences, San Jose, CA, USA) flow cytometer, using the FACSuite Software version 1.0.5.3841 (BD Biosciences, San Jose, CA, USA). To analyze the staining of the cell-surface markers, 10,000 events were counted, and a gate was drawn according to the physical properties (FSC-forward and SSC-scatter) that corresponded to lymphocytes; subsequently, a second gate was drawn according to single cells (FSC-H-forward height vs. FSC-A-forward area). Then, CD4+ cells were selected in a third FSC-CD4 dot, and to determine subsets of activated T cells, a new plot was obtained showing CD4+ vs. CD69+ cells.
